# Determination of Halogens by Ion Chromatography in Edible Mushrooms after Microwave-Induced Combustion for Sample Preparation

**DOI:** 10.1155/2021/6005481

**Published:** 2021-10-31

**Authors:** Gilberto Silva Coelho Junior, Filipe Soares Rondan, Carla Andrade Hartwig, Rafael Francisco Santos, Paola Azevedo Mello, Marcia Foster Mesko

**Affiliations:** ^1^Centro de Ciências Químicas, Farmacêuticas e de Alimentos, Universidade Federal de Pelotas, Capão do Leão 96160-000, RS, Brazil; ^2^Departamento de Química, Universidade Federal de Santa Maria, Santa Maria 97105-900, RS, Brazil

## Abstract

In this study, the microwave-induced combustion (MIC) method was evaluated for the sample preparation of the most consumed mushroom species in Brazil (Champignon, Shiitake, and Shimeji) and further halogen determination by ion chromatography (IC). For this, sample mass, combustion aid mass, and absorbing solution (H_2_O and 50 mmol·L^−1^, 100 mmol·L^−1^, or 150 mmol·L^−1^ NH_4_OH) were evaluated. Bromine and iodine concentrations, determined by IC, were lower than the limits of detection (LODs, Br: 6 mg·kg^−1^ and I: 24 mg·kg^−1^). Inductively coupled plasma mass spectrometry (ICP-MS) was also used for Br and I determination, and the LODs were lower (Br: 0.066 mg·kg^−1^ and I: 0.014 mg·kg^−1^) than those obtained by IC. Concentrations of Cl, obtained by IC, ranged from 523 mg·kg^−1^ to 13053 mg·kg^−1^ with LOD of 40 mg·kg^−1^. In turn, Br and I concentrations, obtained by ICP-MS, ranged from 2.49 mg·kg^−1^ to 5.50 mg·kg^−1^ and from <0.014 mg·kg^−1^ to 0.047 mg·kg^−1^, respectively. Fluorine concentrations, determined by IC, were always lower than LOD (23 mg·kg^−1^). The trueness of the proposed methods was evaluated by recovery tests using standard solutions and a reference material (RM NIST 8435). When using the standard solution, recoveries ranged from 95% to 103% for halogen determination by IC and from 105% to 109% for Br and I determination by ICP-MS. When using the RM, recoveries of 102% for Cl by IC and of 87% and 86% for Br and I by ICP-MS, respectively, were obtained.

## 1. Introduction

Mushrooms are foods that have been appreciated by humanity since ancient times because people believed in their high nutritional value and medicinal potential. Nowadays, mushrooms still have great use as spices in culinary dishes, whose consumption has been increased due to the low calories and high amounts of proteins, vitamins, and minerals [1, 2].

Approximately 2000 edible species of mushrooms are known. However, only about 25 are commercially grown. Among the most cultivated and consumed species in Brazil are the Champignon (*Agaricus*), Shiitake (*Lentinula*), and Shimeji (*Pleurotus*) [3]. Although the production of mushrooms in Brazil is about 12,000 tons year^−1^, the per capita consumption in the country is about 160 g year^−1^. This consumption is low when compared to European countries such as France, Italy, and Germany, which have an annual per capita consumption higher than 2 kg, while Asian countries such as China and South Korea consume more than 8 kg of mushrooms annually [4].

Despite of the high nutritional values of mushrooms, these foods are sources of macronutrients and micronutrients, such as the halogens [5, 6]. In this sense, because some elements can cause health hazards when in inadequate amounts in the organism, it is necessary to know about their concentrations in foods. Thus, in view of their high consumption worldwide, halogens in mushrooms should be monitored.

Among the halogens, bromine (Br) is an essential element that acts as cofactor for the collagen IV formation [7]. However, there are reports in the literature indicating that, depending on the species to which the Br is ingested, it may present carcinogenic effects. Moreover, Br can change the functioning of the thyroid gland, affecting the transport of iodine (I) to the gland, culminating in parallel disorders [8, 9].

Chlorine (Cl) is an important mineral for the human body since it participates in several vital functions. In general, this element controls the anion-cation balance of the blood osmotic pressure and the balance of body water. Also, Cl participates in the nutrient transport across membranes and in regulation of membrane permeability of various tissues, as well as being part of the composition of various enzymes [10].

Fluorine (F) is related with the prevention of dental caries and improves the oral and bone health. However, the excessive ingestion may cause dental and skeletal fluorosis, leading to increased probability of bone fractures [11].

In turn, I is essential for the synthesis of thyroid hormones, regulating a variety of important physiological functions in the body, mainly related to the development, growth, metabolic processes, and cellular oxidation [10, 12, 13].

Thus, Br, Cl, F, and I determination in mushrooms is important to ensure the ingestion of safe levels of these elements. However, only a few studies in the literature performed halogen determination in mushrooms, and most of them use techniques not available in routine laboratories, such as instrumental neutron activation analysis (INAA) and energy dispersive X-ray fluorescence (EDXRF) [6, 14, 15].

In this sense, as alternatives for halogen determination, some analytical techniques have been widely used, such as inductively coupled plasma mass spectrometry (ICP-MS), inductively coupled plasma optical emission spectrometry (ICP-OES), and ion chromatography (IC). Among these analytical techniques, the IC technique with conductivity detection could be highlighted due to its relatively lower cost in relation to analysis, acquisition, and instrument maintenance, and it allows the determination of Br, Cl, F, and I in a single chromatography run, while the determination of F by ICP-OES or ICP-MS is not possible to be performed by conventional ways [16, 17].

To perform analyses by IC technique, the sample must be introduced into the system as a solution. However, the complete digestion of mushrooms is not a simple task, considering their relatively high concentration of ash and crude protein [5, 18]. Furthermore, acid wet digestion methods are not suitable for further determination of halogens [19, 20]. Using acid solution for sample digestion and further halogen determination, there is a risk of analytes loss. Thus, the microwave-induced combustion (MIC) method can be considered a good alternative for the sample preparation of mushrooms, due to the possibility of combustion of organic matter and analytes recovery in a suitable absorbing solution (water or alkaline solutions) [19, 21–27]. Although the MIC method has been successfully applied for the digestion of organic samples for subsequent determination of halogens, there are no reports in the literature of its use for the digestion of mushrooms.

Therefore, in this study, a method for digestion of mushrooms using MIC and further determination of Br, Cl, F, and I by IC was proposed. In addition, Br and I concentrations were also determined by ICP-MS, using the sample preparation conditions selected in the proposed method.

## 2. Materials and Methods

### 2.1. Samples

Mushrooms produced in the state of Rio Grande do Sul, Brazil, were purchased in a local market in natural form. Three species of mushrooms, Champignon (brown and white), Shiitake, and Shimeji, were acquired, totalizing four samples. White Champignon sample was arbitrarily chosen for optimization of the method conditions. The samples that were not used in the optimization tests were further digested using the selected conditions. Prior to the analyses, the samples were individually dried at 65 ± 5°C for 36 h. A reference material (RM), from the National Institute of Standards and Technology (NIST 8435, whole milk powder), was used as a spike to evaluate the trueness of the proposed method.

### 2.2. Instrumentation

Samples and reagents were weighed using an analytical balance (model AY220, Shimadzu, Philippines), with resolution of 0.0001 g. A food processor (model Multi Pro All in One 2, Philco, Brazil) and a conventional oven (model 400/2ND, DeLeo, Brazil) were used to grind and dry the samples, respectively. Ultrapure water (18.3 MΩ·cm) used in this study was obtained from a purification system (model Mega Up, Megapurity, South Korea).

Sample preparation by MIC method was performed using a microwave oven (model Multiwave 3000®, Anton Paar, Austria). This system was equipped with eight high pressure quartz vessels (volume of 80 mL, maximum temperature and pressure of 280°C and 80 bar, respectively) and quartz devices used as sample holder. For the decontamination and further drying of the discs of filter paper and low-density polyethylene (LDPE) films used in the MIC process, an ultrasonic bath (40 kHz, 155 W, model USC-1800 A, Unique, Brazil) and a class 100 laminar flow hood (model CSLH-12, Veco, Brazil) were used. For the pH measurement, digital equipment (model mPA-210, MS Tecnopon, Brazil) was used.

Halogen determination was carried out using an ion chromatograph (model 861 Advanced Compact IC, Metrohm, Switzerland), equipped with an anion self-regeneration suppressor and conductivity detector. Furthermore, Br and I determination was carried out using an inductively coupled plasma mass spectrometer (model NexION 300X, Perkin-Elmer, Canada) equipped with a concentric nebulizer (Meinhard Associates, USA), a cyclonic spray chamber (Glass Expansion Inc., Australia), and a quartz torch with a quartz injector tube (2 mm i.d.). The operating conditions for the IC and ICP-MS are shown in Table 1.

### 2.3. Reagents

All solutions and dilutions were prepared using ultrapure water, and all reagents were of analytical grade or higher purity. Solutions of NH_4_OH used as absorbing solution were prepared from commercial NH_4_OH solution (27% m/m NH_3_, Synth, Brazil). Ammonium nitrate solution (6 mol L^−1^), used as igniter and/or as combustion aid by MIC, was prepared by dissolution of the solid reagent (Merck, Germany) in water.

Decontamination of the quartz vessels and holders used in MIC method was performed using 6 mL of 65% (m/m) HNO_3_ (Vetec, Brazil) followed by 6 mL of ultrapure water, according to previous works [28–30]. Small discs (approximately 12 mg, 15 mm of diameter) of filter paper (0.5% ash content, Qualy, J Prolab, Brazil) were used in combustion step, and LDPE films (80 × 80 mm) were used to wrap the samples [31].

The mobile phase used during IC analyses (3.2 mmol L^−1^ Na_2_CO_3_ and 1.0 mmol L^−1^ NaHCO_3_) was prepared by the dissolution of corresponding salts (Synth) in water. Sulfuric acid solution (0.2 mol L^−1^), prepared from 95 to 99% (m/m) H_2_SO_4_ (Merck), was used for sodium suppression in the system regeneration.

Argon with purity of 99.996% (White Martins, Brazil) was used for plasma generation and nebulization and as auxiliary gas for Br and I determination by ICP-MS. The purity of the O_2_ used for the vessels pressurization in MIC method was 99.6% (White Martins).

For the analyte determination by IC or ICP-MS, as well as recoveries tests, solutions were prepared by dilution of stock solutions of Br^−^, Cl^−^, F^−^, and I^−^ which were prepared by dissolution of the respective salts of KBr (Merck), KCl (Synth), NaF (Synth), and KI (Merck), respectively, in ultrapure water. The calibration ranges for IC determination were from 0.1 mg L^−1^ to 1.0 mg L^−1^ for Br^−^, Cl^−^, F^−^, and I^−^. For ICP-MS determination, the calibration range was from 1 *µ*g L^−1^ to 10 *µ*g L^−1^ for Br and 0.1 *µ*g L^−1^ to 1.0 *µ*g L^−1^ for I.

### 2.4. Sample Preparation by the MIC Method

In initial studies by MIC, powdered sample of White Champignon was weighed and wrapped with a LDPE film. The LDPE film was then sealed by heating, and the excess of film was removed (film mass final used was approximately 30 mg). Aiming at better decompositions of the samples, 6 mol L^−1^ NH_4_NO_3_ was mixed with the samples, taking into account that preview study in the literature used this reagent as combustion aid [31]. A small disc of filter paper was placed on the base of a quartz holder. The filter paper was moistened with 50 *µ*L of 6 mol L^−1^ NH_4_NO_3_ (igniter solution) and the wrap containing the sample was placed on the paper. The quartz holder was introduced inside the quartz vessel previously charged with 6 mL of absorbing solution. Ultrapure water and 50 mmol L^−1^ to 150 mmol L^−1^ NH_4_OH solutions were evaluated for analytes absorption. After closing, the vessels were positioned on the rotor, pressurized with 20 bar of O_2_, and the rotor was placed inside the microwave oven.

The microwave heating program used for sample preparation by MIC was 1400 W for 5 min (combustion and reflux steps) and, in the sequence, 0 W was applied for 20 min (cooling step) [31, 32]. After the procedure, the gases and vapors of each vessel were released and digests were transferred to volumetric flasks, being diluted with ultrapure water up to 25 mL. The analytes determination was performed by the analytical techniques previously described.

The trueness of the optimized proposed method was evaluated by recovery tests using standard solutions containing all analytes and by analysis of a RM for Br and I. The recovery tests were performed by the addition of a standard solution to the sample (selected mass) before MIC digestion. Thus, the solution was prepared considering that the added volume represented 50% of the concentrations of the analytes present in the samples. For the analytes not detected in the samples, 1.5 times the limit of quantification (LOQ) were added. Moreover, a similar recovery test was performed by combustion of a mixture containing 50 mg of RM NIST 8435 and 500 mg of the mushroom sample. After, the proposed method was applied for other samples evaluated in this study. The halogen determination in digests from MIC was performed by IC technique. In addition, the same digests were also analyzed by ICP-MS for Br and I determination.

All results were statistically evaluated by one-way analysis of variance (ANOVA) followed by the Tukey test or Student's *t*-test (confidence level of 95%) using GraphPad InStat version 3.00 computer software package (GraphPad, USA).

## 3. Results and Discussion

### 3.1. Evaluation of the Maximum Mass of Mushroom Digested by MIC

For this evaluation, water was used as absorbing solution considering that the focus of this study was only to verify the aspect of the sample after digestion. Thus, initially, 200 mg of dry sample mass was digested by MIC, and after each subsequent test, 100 mg of sample was increased. During this evaluation, in addition to the aspect of the digests, the maximum pressure reached by the system was monitored. When evaluating sample masses from 200 mg to 400 mg, colorless digests and clean quartz holders and vessels were observed, indicating a suitable digestion. However, for digestion of 500 mg of sample, an unsuitable digestion was obtained, characterized by the presence of residues in the sample holder and a yellowish solution. As form of increasing the sample mass efficiently digested by MIC, the use of a combustion aid was evaluated. Thus, 50 *µ*L of 6 mol L^−1^ NH_4_NO_3_ (combustion igniter solution) was mixed to 500 mg of sample and a suitable digestion was obtained. Beyond, when we performed this evaluation, the maximum pressure observed in the system was 39 bar, which correspond to 49% of the maximum pressure (80 bar) recommended by manufacturer of the microwave oven. On the other hand, when higher sample mass (600 mg with 50 *µ*L of 6 mol L^−1^ NH_4_NO_3_) was evaluated, unsuitable digestion was obtained. It is important to mention that the use of a high sample is important to obtain better LODs and LOQs. In this sense, the sample mass of 500 mg mixed with 50 *µ*L of 6 mol L^−1^ NH_4_NO_3_ was selected as better condition for digestion of mushrooms by the MIC method.

### 3.2. Limits of Detection and Quantification

Prior to the evaluation of the absorbing solution for MIC method, the LODs and LOQs calculations were performed for each analyte and method. These limits were used to define the concentrations to be added in the samples in the recovery tests for those elements that were below the LOQ in the evaluated samples. In this sense, considering that the reagent blank values were used for the calculations, this study aimed to verify the influence caused by different absorbing solutions in the reagent blanks.

For LOD and LOQ calculation, the average of the blanks (10 readings) for each analyte plus 3 times the standard deviation (for LOD) or 10 times the standard deviation (for LOQ) were considered [33]. Furthermore, in the calculation, the dilution factor, the final volume of the digests, and the sample mass were considered. In this sense, the reagent blanks obtained after the sample preparation, using different absorbing solutions (ultrapure water and 50 mmol L^−1^, 100 mmol L^−1^, and 150 mmol L^−1^ NH_4_OH), were analyzed. For determination by IC, only the peak related to the retention time of Cl was detected in the chromatograms. On the other hand, an interference was observed near to the retention time of F, while Br and I were not detected in the blanks because these elements were in concentrations below that detectable in the chromatograms.

Taking into account that the average of the blanks was used for LOD calculation for F, dilutions of the blanks of digestion using ultrapure water as absorbing solution were carried out with the addition of a known concentration of this element in solution (0.05 mg L^−1^) until the absence of interference and obtaining an adequate recovery. In view of this, it was observed that, after a dilution factor of 5 times, suitable recovery and no interference were observed. Thus, this dilution factor, as well as the results obtained from the analyses previously mentioned, was considered for LOD and LOQ calculation. The LOD and LOQ for F were, respectively, 23 mg kg^−1^ and 30 mg kg^−1^. For Br and I, LODs were performed considering the baseline noise for the retention time of these elements. Thus, a LOD of 6 mg kg^−1^ and 24 mg kg^−1^ and a LOQ of 7 mg kg^−1^ and 61 mg kg^−1^ for Br and I, respectively, were obtained.

**Figure 1:**
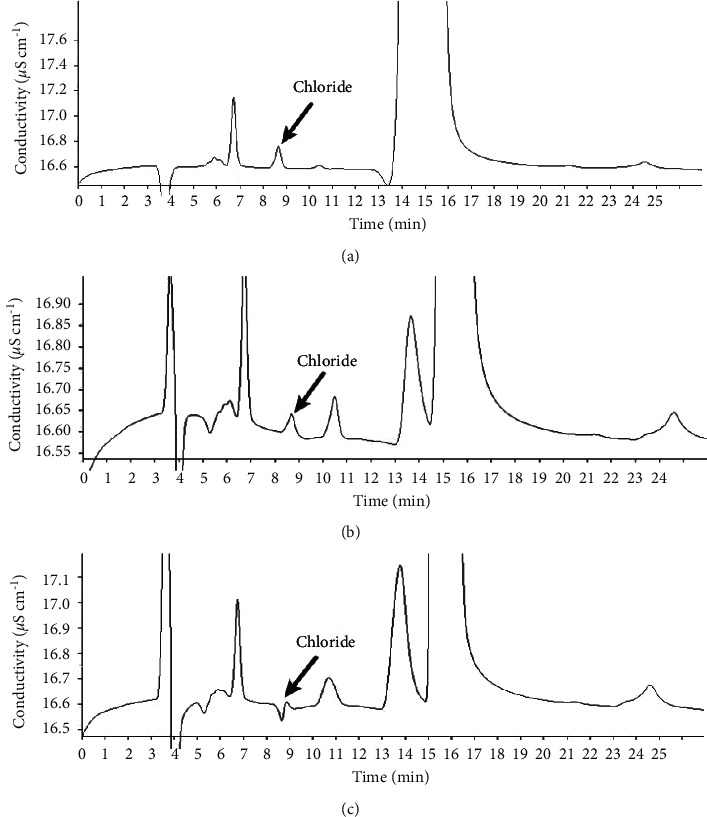
Chromatograms of the blanks using (a) ultrapure water, (b) 50 mmol L^−1^ NH_4_OH, and (c) 100 mmol L^−1^ NH_4_OH as absorbing solution.

On the other hand, interference was observed in the Cl retention time, possibly due to the use of 100 mmol L^−1^ NH_4_OH as absorbing solution, as shown in Figure 1. Thus, dilutions of the blanks were carried out with the addition of a known concentration of Cl (0.1 mg L^−1^) until the absence of interference and an adequate recovery; therefore, a dilution of 4 times was needed. In this sense, different values of LOD and LOQ for Cl were obtained, as shown in Table 2. Based on Table 2, when using 100 mmol L^−1^ NH_4_OH solution, it was possible to observe an increase of about 4 times for LOD and LOQ.

The LODs and LOQs for Br and I by ICP-MS were considerably lower than those obtained by IC, as can be observed in Table 2, ranging from 88 times to 3000 times lower than those obtained by IC, using the different absorbing solutions evaluated. This decrease in LOD values demonstrates that ICP-MS has a greater sensitivity for determination of these elements. In addition, it is worth mentioning that when increasing the concentration of the absorbing solution, the LODs for Br also increased, possibly because the solution has better absorption characteristics of the analyte. On the other hand, for I, this behavior was not observed, since in increasing the concentration of the absorbing solution, no significant increases were observed in the LODs or LOQs.

### 3.3. Evaluation of the Absorbing Solutions for Halogens

In order to obtain accurate results, different absorbing solutions were evaluated to choose the better solution for halogen absorption. Thus, for IC analysis, ultrapure water and NH_4_OH solutions (50 mmol L^−1^ and 100 mmol L^−1^) were evaluated and, for ICP-MS analysis, ultrapure water and NH_4_OH solutions (50 mmol L^−1^, 100 mmol L^−1^, and 150 mmol L^−1^) were evaluated. The absorbing solutions used in this study were selected considering previous studies that recommend these solutions for halogens [30, 32, 34]. For this evaluation, recovery tests were performed by addition of a known analyte concentration (corresponding to 50% of the obtained concentration in the sample used for optimization) in the sample for each absorbing solution evaluated. The standard solution was added in the samples prior the digestion by MIC method.

**Figure 2:**
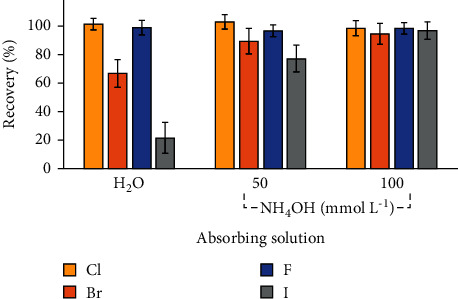
Recoveries obtained by IC for chlorine after the addition of 50% of the concentration obtained in the samples and for bromine, fluorine, and iodine after the addition of 1.5 times the concentration of the LOQs for each element (*n* = 3).

During the determination of halogens by IC, it was observed that only Cl could be determined, while Br and I were below the LOD. For F, interference was observed in the same retention time of this analyte, making it necessary to perform dilutions to enable the determinations. Taking into account these facts, for Br, F, and I, the recovery tests were performed by the addition of known concentrations of these elements (1.5 times the concentration obtained for the LOQs of each analyte (Section 3.2). The recoveries for all analytes are shown in Figure 2.

As shown in Figure 2, suitable recoveries for Cl and F in all absorbing solutions evaluated were obtained, with recoveries ranging from 97% to 103% and relative standard deviations (RSDs) lower than 6%. In this sense, it is important to point out that no significant differences (ANOVA, confidence level of 95%) were observed for Cl and F recoveries using all absorbing solutions. However, for Br and I, unsuitable recoveries were obtained, 67% and 89% for Br and 22% and 77% for I, when using, respectively, ultrapure water and 50 mmol L^−1^ NH_4_OH. In contrast, suitable recoveries (95% for Br and 97% for I) were obtained using 100 mmol L^−1^ NH_4_OH solution, with RSDs lower than 8%. Moreover, when evaluating the pH of the diluted digests, values below 5.8 were observed for ultrapure water and 50 mmol L^−1^ NH_4_OH, while when 100 mmol L^−1^ NH_4_OH was used a pH of 7.6 was observed. It is important to point out that alkaline solutions lead to better halogen stabilization, as demonstrated in other studies [28, 35].

In view of the suitable recoveries for all halogens by IC, the 100 mmol L^−1^ NH_4_OH was selected as method condition. This fact corroborates with some studies reported in the literature, which mention that 100 mmol L^−1^ NH_4_OH is a suitable solution for further halogen determination by IC [26, 31, 36].

However, aiming at lower reagent consumption, as well as a higher practicality in the sample preparation, it is important to highlight that the use of ultrapure water allowed a suitable Cl and F absorption, which can be noted in the suitable recoveries for these analytes. Moreover, ultrapure water as absorbing solution showed lower LODs for Cl regarding to the other solutions evaluated.

**Figure 3:**
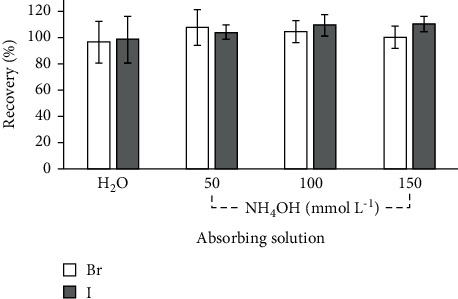
Recoveries obtained by ICP-MS for bromine and iodine in mushrooms digested by MIC after the addition of 75% of bromine concentration and 2 times the LOQ for iodine (*n* = 3).

Taking into account that it was not possible to determine Br and I in the samples by IC due to its low concentration, this determination was performed by ICP-MS. The choice of this technique was due its higher sensitivity for Br and I when compared to the IC analysis [17]. Moreover, when comparing the LODs between both techniques, it is possible to observe a considerable difference in the values. Thus, a recovery test with the addition of a known concentration of Br and I in the sample prior to the digestion by MIC was performed. The concentration of the solution added in the sample corresponded to 75% of the concentration of Br in the analyzed samples and 2 times the LOQ obtained for I by ICP-MS, in view that concentration for I, in the analyzed sample, was below the LOQ. The results of the recovery tests are shown in Figure 3.

As shown in Figure 3, suitable recoveries for Br and I were observed using all absorbing solutions, ranging from 97% to 108% for Br and from 98% to 110% for I. When evaluating the different absorbing solutions, no significant differences (ANOVA, confidence level of 95%) were observed for both analytes.

Moreover, the pH values of the digests were evaluated and only 100 mmol L^−1^ and 150 mmol L^−1^ NH_4_OH solutions presented slightly alkaline values (7.6 and 8.2, respectively). Thus, in view of the suitable recoveries, 100 mmol L^−1^ NH_4_OH solution was selected as the most suitable condition to absorb Br and I for subsequent analysis by ICP-MS. Moreover, it is important to mention that though the solution of 150 mmol L^−1^ NH_4_OH did not present significant differences regarding the 100 mmol L^−1^ NH_4_OH for Br and I concentrations, the lower concentration was selected in view of the lower reagent consumption.

For the trueness evaluation of the proposed method for Cl determination by IC and Br and I determination by ICP-MS, the RM NIST 8435 was also used. A suitable trueness for Cl by IC was observed, with recoveries of 102% between spiked value (843 mg kg^−1^) and the recovered value (856 ± 6 mg kg^−1^). However, recoveries of 87% for Br and 86% for I by ICP-MS were observed, where the values spiked were 2.00 mg kg^−1^ for Br and 0.23 mg kg^−1^ for I, while the recovered values were 1.74 ± 0.09 mg kg^−1^ for Br and 0.20 ± 0.01 mg kg^−1^ for I. The lower recoveries for these analytes can be related to the high standard deviation (SD) presented by the RM certificate. The RSDs for the reference concentrations of Br and I in the RM NIST 8435 are 50% and 17%, respectively. Moreover, though the RM present values for Br, F, and I, its determination was not possible by IC due the lower concentration of these elements in the material.

### 3.4. Determination of Halogens in Mushrooms

After the choice of the more suitable sample preparation conditions and the accuracy evaluation of the proposed methods, the analysis of the other mushrooms species was performed. The results regarding to determination of Br, Cl, F, and I by IC in the samples are shown in Table 3.

As observed in Table 3, using IC, only Cl concentration was possible to be determined in the samples evaluated, in view that Br, F, and I concentrations were below the LODs. For Cl, a large range of concentration (from 523 mg kg^−1^ to 13053 mg kg^−1^) was observed, in which the Champignon species presented the higher values. The variation in Cl concentration possibly is related to the nutrients added in the cultivation of different species of mushrooms [5].

According to the daily intake of Cl recommended by the Institute of Medicine of the National Academies of Science (Washington, USA), an adult who is between 19 and 50 years old should receive an intake of 2300 mg of Cl daily with the goal of obtaining a good functioning level of the body [37]. Although the values for Cl in the samples evaluated are considered high, it is important to emphasize that the concentration is related to dry base. Thus, when considering the dry mushrooms, about 177 g of White Champignon provides the recommended daily intake of Cl. However, when Shimeji mushroom is ingested, about 4.4 kg is necessary to provide the daily recommended intake of Cl. It is important to point out that these correlations are based on a daily diet of only mushroom.

In Table 4, the Br and I concentrations determined by ICP-MS after digestion by the MIC method of the different species of mushrooms are shown.

As shown in Table 4, it is possible to observe that Br concentration in all the samples evaluated was higher than the LOD (0.066 mg kg^−1^), varying in a small concentration range (2.49 mg kg^−1^ to 5.50 mg kg^−1^). In this context, taking into account that Br has no daily intake recommendation, it is not possible to estimate if the concentration of this element is suitable for the human consumption. For I, it was possible to observe that only one sample (White Champignon) showed concentration below the LOD (0.014 mg kg^−1^). However, for the other samples, a small variation in the I concentration was observed (from 0.019 mg kg^−1^ to 0.047 mg kg^−1^).

According to the daily intake of I recommended by the Third National Health and Nutrition Examination Survey (Georgia, USA), an adult should have an intake of 140 *µ*g of I daily with the goal of obtaining a good functioning level of the body [37]. Taking into account that the values for I in the samples evaluated are low, when considered the intake only of dry mushrooms, about 3 kg of Shimeji (sample with higher concentration of I) would be needed to provide the daily recommended intake of I.

The results obtained for Br, Cl, and I in this study were compared to those reported in the literature for mushrooms of different species [6, 14, 15, 38]. Based on the concentration ranges for these elements presented in Table 5, it can be observed that the values for Br, Cl, and I are within those reported in the literature. These variations between the values can be related to the different species of mushrooms evaluated in the works, as well as their different origins and type of cultivation.

## 4. Conclusion

In view of the results obtained during this study, it was possible to conclude that the developed method was suitable for the determination of halogens in mushrooms after decomposition by MIC and subsequent determination by IC, as well as determination of Br and I by ICP-MS. In addition, the proposed method showed a high sample throughput. Furthermore, this method provided the use of diluted reagents, reducing reagent consumption during the analyses and compatible digests for both the determination techniques.

In view of the application of the method using IC, it was not possible to determine Br, F, and I because the concentrations were below the LODs. On the other hand, using ICP-MS it was possible to determine Br in all the samples evaluated and I in the majority of the samples, and a small variation in the concentration of these analytes was observed between the different species of mushroom.

Moreover, it should be noted that although it was not possible to perform the determination of some elements by IC, due to the low concentrations in the mushrooms, the developed method presented good accuracy for all the elements. Finally, it is possible to highlight the potential of the use of MIC for the sample preparation of mushroom, the use of IC for the determination of halogens, and the ICP-MS especially for the determination of Br and I, as well as the compatibility of digests obtained by MIC with different determination techniques.

## Figures and Tables

**Table 1:** Operational conditions for halogen determination by IC and by ICP-MS.

Parameter	Condition
IC	
Stationary phase	Poly(vinyl alcohol) with quaternary ammonium groups
Mobile phase (mmol L^−1^)	Na_2_CO_3_ (3.2)/NaHCO_3_ (1.0)
Suppression solution (mol L^−1^)	H_2_SO_4_ (0.2)
Flow rate (mL min^−1^)	0.7
Sample loop (*µ*L)	20
Column	Metrosep A Supp 5 (150 mm x 4 mm i.d.)
Guard column	Metrosep A Supp 4/5 Guard (5 mm × 4 mm i.d.)
Detection	Conductivity

ICP-MS	
Radiofrequency potency (W)	1300
Plasma argon flux (L min^−1^)	18
Auxiliary argon flux (L min^−1^)	1.20
Nebulizer argon flux (L min^−1^)	0.95
Sample uptake rate (mL min^−1^)	0.7
Spray chamber	Cyclonic
Nebulizer	Concentric
Sampler and skimmer cones	Pt
Ion lens	Auto lens “On”
Isotopes (m/z)	^79^Br, ^127^I

**Table 2:** Limits of detection and quantification for chlorine by IC and for bromine and iodine by ICP-MS, using different absorbing solutions for sample preparation of mushrooms (500 mg) by MIC.

Absorbing solution	LOD (mg kg^−1^)			LOQ (mg kg^−1^)		
	Chlorine	Bromine	Iodine	Chlorine	Bromine	Iodine
Ultrapure water	9	0.005	0.008	17	0.019	0.012
50 mmol L^−1^ NH_4_OH	10	0.040	0.014	18	0.069	0.016
100 mmol L^−1^ NH_4_OH	40	0.066	0.014	74	0.096	0.015
150 mmol L^−1^ NH_4_OH	Nd^*∗*^	0.068	0.014	Nd^*∗*^	0.099	0.016

^*∗*^Not determined.

**Table 3:** Concentration of halogens in mushrooms (dry base; 500 mg) after digestion by the MIC method using 100 mmol L^−1^ NH_4_OH as absorbing solution and determination by IC (mean ± standard deviation; *n* = 3).

Mushroom	Concentration (mg kg^−1^)			
	Bromine	Chlorine	Fluorine	Iodine
Brown Champignon	<6^*∗*^	9861 ± 1017	<23^*∗*^	<24^*∗*^
White Champignon	<6^*∗*^	13053 ± 216	<23^*∗*^	<24^*∗*^
Shiitake	<6^*∗*^	529 ± 77	<23^*∗*^	<24^*∗*^
Shimeji	<6^*∗*^	523 ± 37	<23^*∗*^	<24^*∗*^

^*∗*^Limit of detection.

**Table 4:** Concentration of bromine and iodine in mushrooms (dry base; 500 mg) after digestion by the MIC method using 100 mmol L^−1^ NH_4_OH as absorbing solution and determination by ICP-MS (mean ± standard deviation; *n* = 3).

Mushroom	Concentration (mg kg^−1^)	
	Bromine	Iodine
Brown Champignon	5.50 ± 0.37	0.040 ± 0.002
White Champignon	4.31 ± 0.09	<0.014^*∗*^
Shiitake	2.49 ± 0.10	0.019 ± 0.001
Shimeji	3.40 ± 0.48	0.047 ± 0.007

^*∗*^Limit of detection.

**Table 5:** Concentration range for bromine, chlorine, and iodine in mushrooms reported in the literature and in the samples analyzed in this study.

Reference	Concentration (mg kg^−1^)		
	Bromine	Chlorine	Iodine
[14]	0.24–177	74–26774	Nd
[38]	Nd	Nd	0.044–0.43
[6]	0.13–2.7	Nd	Nd
[15]	4.2–27.2	Nd	Nd
This study	2.49–5.50	523–13053	<0.014^*∗*^–0.047

^*∗*^Limit of detection; Nd: not determined.

## Data Availability

The data used to support the findings of this study are available at Theses & Dissertations Catalog (CAPES), Brazil (https://sucupira.capes.gov.br/sucupira/public/consultas/coleta/trabalhoConclusao/viewTrabalhoConclusao.jsf?popup=true&id_trabalho=5685136).

## References

[1] Rácz L., Papp L., Prokai B., Kovács Z. (1996). Trace element determination in cultivated mushrooms: an investigation of manganese, nickel, and cadmium intake in cultivated mushrooms using ICP atomic emission. *Microchemical Journal*.

[2] Wani B. A., Bodha R. H., Wani A. H. (2010). Nutritional and medicinal importance of mushrooms. *Journal of Medicinal Plants Research*.

[3] Urben A. F., Oliviera H. C. B., Vieira W., Correia M. J., Uriartt A. H. (2001). *Produção de cogumelos por meio de tecnologia chinesa modificada*.

[4] ANPC Associação Nacional dos Produtores de Cogumelos (2013). *Cogumelos no brasil*.

[5] Chang S., Miles P. G. (2004). *Mushrooms Cultivation, Nutritional Value, Medicinal Effect, and Environmental Impact*.

[6] Moura P. L. C., Maihara A., Castro L. P., Figueira R. C. L. (2007). Essential trace elements in edible mushrooms by neutron activation analysis. *International Nuclear Atlantic Conference – INAC*.

[7] McCall A. S., Cummings C. F., Bhave G., Vanacore R., Page-McCaw A., Hudson B. G. (2014). Bromine is an essential trace element for assembly of collagen IV scaffolds in tissue development and architecture. *Cell*.

[8] Deangelo A. B., George M. H., Kilburn S. R., Moore T. M., Wolf D. C. (1998). Carcinogenicity of potassium bromate administered in the drinking water to male B6C3F1 mice and F344/N rats. *Toxicologic Pathology*.

[9] Vobecky M., Babicky A., Lener J. (1996). Effect of increased bromide intake on iodine excretion in rats. *Biological Trace Element Research*.

[10] WHO (1996). *Trace Elements in Human Nutrition and Health*.

[11] Preed V. R. (2015). *Fluorine Chemistry, Analysis, Function and Effects*.

[12] WHO (2007). *Assessment of Iodine Deficiency Disorders and Monitoring Their Elimination - a Guide for Programme Managers*.

[13] Zimmermann M. B., Jooste P. L., Pandav C. S. (2008). Iodine-deficiency disorders. *The Lancet*.

[14] Randa Z., Kucera J. (2004). Trace elements in higher fungi (mushrooms) determined by activation analysis. *Journal of Radioanalytical and Nuclear Chemistry*.

[15] Turhan Ş., Zararsız A., Karabacak H. (2010). Determination of element levels in selected wild mushroom species in Turkey using non-destructive analytical techniques. *International Journal of Food Properties*.

[16] Bu X., Wang T., Hall G. (2003). Determination of halogens in organic compounds by high resolution inductively coupled plasma mass spectrometry (HR-ICP-MS). *Journal of Analytical Atomic Spectrometry*.

[17] Mello P. A., Barin J. S., Duarte F. A. (2013). Analytical methods for the determination of halogens in bioanalytical sciences: a review. *Analytical and Bioanalytical Chemistry*.

[18] Manzi P., Gambelli L., Marconi S., Vivanti V., Pizzoferrato L. (1999). Nutrients in edible mushrooms: an inter-species comparative study. *Food Chemistry*.

[19] La Rosa Novo D., Pereira R. M., Henn A. S., Costa V. C., Moraes Flores E. M., Mesko M. F. (2019a). Are there feasible strategies for determining bromine and iodine in human hair using interference-free plasma based-techniques?. *Analytica Chimica Acta*.

[20] Todolí J.-L., Mermet J.-M. (1999). Acid interferences in atomic spectrometry: analyte signal effects and subsequent reduction. *Spectrochimica Acta Part B: Atomic Spectroscopy*.

[21] Coelho Junior G. S., Hartwig C. A., Toralles I. G. (2017). Determination of Cl and S in edible seaweed by ion chromatography after decomposition by microwave-induced combustion. *Revista Virtual de Química*.

[22] Mello P. d. A., Giesbrecht C. K., Alencar M. S. (2008). Determination of sulfur in petroleum coke combining closed vessel microwave-induced combustion and inductively coupled plasma-optical emission spectrometry. *Analytical Letters*.

[23] de Mello J. E., Novo D. L. R., Coelho Junior G. S., Mesko M. F., Mesko M. F. (2020). A green analytical method for the multielemental determination of halogens and sulfur in pet food. *Food Analytical Methods*.

[24] Mesko M. F., Balbinot F. P., Scaglioni P. T., Nascimento M. S., Picoloto R. S., da Costa V. C. (2020). Determination of halogens and sulfur in honey: a green analytical method using a single analysis. *Analytical and Bioanalytical Chemistry*.

[25] Mesko M. F., Costa V. C., Picoloto R. S., Bizzi C. A., Mello P. A. (2016a). Halogen determination in food and biological materials using plasma-based techniques: challenges and trends of sample preparation. *Journal of Analytical Atomic Spectrometry*.

[26] Mesko M. F., Toralles I. G., Hartwig C. A. (2016b). Bromine and iodine contents in raw and cooked shrimp and its parts. *Journal of Agricultural and Food Chemistry*.

[27] da Silva S. V., Picoloto R. S., Flores E. M. M., Wagner R., dos Santos Richards N. S. P., Barin J. S. (2016). Evaluation of bromine and iodine content of milk whey proteins combining digestion by microwave-induced combustion and ICP-MS determination. *Food Chemistry*.

[28] Costa V. C., Picoloto R. S., Hartwig C. A., Mello P. A., Flores E. M. M., Mesko M. F. (2015). Feasibility of ultra-trace determination of bromine and iodine in honey by ICP-MS using high sample mass in microwave-induced combustion. *Analytical and Bioanalytical Chemistry*.

[29] Novo D. L. R., Mello J. E., Rondan F. S., Henn A. S., Mello P. A., Mesko M. F. (2019b). Bromine and iodine determination in human saliva: challenges in the development of an accurate method. *Talanta*.

[30] Rondan F. S., Hartwig C. A., Novo D. L. R. (2018). Ultra-trace determination of bromine and iodine in rice by ICP-MS after microwave-induced combustion. *Journal of Food Composition and Analysis*.

[31] Toralles I. G., Coelho G. S., Costa V. C., Cruz S. M., Flores E. M. M., Mesko M. F. (2017). A fast and feasible method for Br and I determination in whole egg powder and its fractions by ICP-MS. *Food Chemistry*.

[32] Rondan F. S., Coelho Junior G. S., Henn A. S., Muller E. I., Mesko M. F., Mesko M. F. (2019). A versatile green analytical method for determining chlorine and sulfur in cereals and legumes. *Food Chemistry*.

[33] http://www.eurachem.org.

[34] Pereira R. M., Costa V. C., Hartwig C. A. (2016). Feasibility of halogen determination in noncombustible inorganic matrices by ion chromatography after a novel volatilization method using microwave-induced combustion. *Talanta*.

[35] Mesko M. F., Toralles I. G., Coelho J. (2020). Ion chromatography coupled to mass spectrometry as a powerful technique for halogens and sulfur determination in egg powder and its fractions. *Rapid Communications in Mass Spectrometry*.

[36] Silva J. S., Diehl L. O., Frohlich A. C. (2017). Determination of bromine and iodine in edible flours by inductively coupled plasma mass spectrometry after microwave-induced combustion. *Microchemical Journal*.

[37] Otten J. J., Hellwig J. P., Meyers L. D. (2006). *Dietary Reference Intakes: The Essential Guide to Nutrient Requirements*.

[38] Haldimann M., Alt A., Blanc A., Blondeau K. (2005). Iodine content of food groups. *Journal of Food Composition and Analysis*.

